# A tumor-promoting mechanism mediated by retrotransposon-encoded reverse transcriptase is active in human transformed cell lines

**DOI:** 10.18632/oncotarget.1403

**Published:** 2013-10-14

**Authors:** Ilaria Sciamanna, Alberto Gualtieri, Cristina Cossetti, Emanuele Felice Osimo, Manuela Ferracin, Gianfranco Macchia, Eleonora Aricò, Gianni Prosseda, Patrizia Vitullo, Tom Misteli, Corrado Spadafora

**Affiliations:** ^1^ Istituto Superiore di Sanità, Viale Regina Elena 299, Rome, Italy.; ^2^ Laboratory for Technologies of Advanced Therapies (LTTA) and Department of Morphology, Surgery and Experimental Medicine, University of Ferrara, Ferrara, Italy.; ^3^ Department of Biology and Biotechnology “Charles Darwin”, Sapienza University, Rome, Italy.; ^4^ National Cancer Institute, NIH, Bethesda MD, USA.; ^5^ Department of Experimental Medicine and Surgery - University of Rome “Tor Vergata”, Rome, Italy.

**Keywords:** LINE-1 retrotransposons, reverse transcriptase, transcriptome, miRNAs, DNA:RNA hybrids, cancer genome, reverse transcriptase inhibitor

## Abstract

LINE-1 elements make up the most abundant retrotransposon family in the human genome. Full-length LINE-1 elements encode a reverse transcriptase (RT) activity required for their own retrotranpsosition as well as that of non-autonomous Alu elements. LINE-1 are poorly expressed in normal cells and abundantly in cancer cells. Decreasing RT activity in cancer cells, by either LINE-1-specific RNA interference, or by RT inhibitory drugs, was previously found to reduce proliferation and promote differentiation and to antagonize tumor growth in animal models. Here we have investigated how RT exerts these global regulatory functions.

We report that the RT inhibitor efavirenz (EFV) selectively downregulates proliferation of transformed cell lines, while exerting only mild effects on non-transformed cells; this differential sensitivity matches a differential RT abundance, which is high in the former and undetectable in the latter. Using CsCl density gradients, we selectively identify Alu and LINE-1 containing DNA:RNA hybrid molecules in cancer but not in normal cells. Remarkably, hybrid molecules fail to form in tumor cells treated with EFV under the same conditions that repress proliferation and induce the reprogramming of expression profiles of coding genes, microRNAs (miRNAs) and ultraconserved regions (UCRs). The RT-sensitive miRNAs and UCRs are significantly associated with Alu sequences.

The results suggest that LINE-1-encoded RT governs the balance between single-stranded and double-stranded RNA production. In cancer cells the abundant RT reverse-transcribes retroelement-derived mRNAs forming RNA:DNA hybrids. We propose that this impairs the formation of double-stranded RNAs and the ensuing production of small regulatory RNAs, with a direct impact on gene expression. RT inhibition restores the ‘normal’ small RNA profile and the regulatory networks that depend on them. Thus, the retrotransposon-encoded RT drives a previously unrecognized mechanism crucial to the transformed state in tumor cells.

## INTRODUCTION

Evidence that the genome is pervasively transcribed, including in its non-coding component, is now an established finding [1] and is changing our views of global mechanisms of regulation of genome function [2]. It is emerging that non-protein coding sequences have key roles in complex pathways regulating the genome functional profile and are often dysregulated in cancer.

Mobile retroelements, including retrotransposons and endogenous retroviruses, make up as much as 45% of the human genome [3]. In contrast with traditional views that considered them to be functionally inert, parasitic burden of eukaryotic genomes, retroelements are in fact extensively transcribed, harboring about 30% of all human transcription start sites [4]. Alu and LINE-1 elements are the largest non-autonomous and autonomous retrotransposon families, accounting for about 10% and 17% of the human genome, respectively [5]. Full-length LINE-1 elements encode their own reverse transcriptase (RT) enzyme, responsible for retrotranscription and, hence, RNA-dependent mobilization of both LINE-1 elements themselves and of non-autonomous Alus. Both families influence the transcriptional output of the genome and growing studies highlight roles of both families in tumorigenesis [6-8].

Alu and LINE-1 families display differentially modulated patterns of expression [4 and references therein]. Alus are stress-responsive elements transcribed by Pol III and show upregulated expression in response to various environmental stimuli (reviewed in 9). Alu-derived RNA is exported to the cytoplasm and processed into small cytoplasmic RNAs (scAlu RNAs) that target matching Alu-containing mRNAs by specific base-pairing, thus decreasing their stability [10,11]. Other proposed mechanisms for Alu roles in gene regulation include nuclear retention of Alu-embedded mRNAs, alternative splicing and repression of translation into protein products [9].

LINE-1 elements are transcribed by Pol II. They provide a source of small RNAs derived from bidirectional transcripts and endowed with regulatory functions [12]. They act as key players in embryonic development [13] and as causative agents in numerous diseases, including cancer [for a review see 14]. Indeed, LINE-1s are highly expressed during embryogenesis [13] and in embryonic stem cells [15], as other retroelement families, indicating that early embryos represent a permissive environment for both reverse transcription [16] and retrotransposition [17]. In contrast, both LINE-1 transcript abundance and retrotransposition are reduced in terminally differentiated non-dividing cells [18]. Retroelements are however highly active in cancer cells and tissues [for a review see 6]. In recent work using a well characterized mouse model of breast cancer progression, i.e. the MMTV murine strain [19], we have been able to pinpoint overexpression of LINE- 1 elements and of the RT activity which they encode, as well as LINE-1 copy number amplification in early stages of breast cancer progression, suggesting that LINE-1 activity can be regarded as an early cancer marker [20].

Previous work in our laboratory showed that LINE-1-encoded RT activity is crucial for control of cell proliferation, differentiation and tumor progression [reviewed in 21]. Indeed, RT downregulation via RNA interference (RNAi) to active LINE-1 element was associated with redifferentiation, decreased proliferation and reduced tumorigenic potential of transformed cells [22, 23]. RT inhibitors are powerful tools to probe RT function. Efavirenz (EFV), a non-nucleoside RT inhibitor originally designed to target the HIV-encoded RT, has a demonstrated ability to inhibit the endogenous cellular RT [24]; remarkably, EFV elicits the same cancer reversal phenotypes [22, 23, 25] observed after RNAi silencing of active LINE-1 elements [23, 26]. Similar effects were reported in human cancer cell lines after treatment with nevirapine, another non-nucleoside RT inhibitor [27] or with the nucleosidic RT inhibitor abacavir [28 and references herein]. Furthermore, EFV has anti-cancer efficacy in vivo and antagonizes the development of human tumors xenografted in nude mice [23]. These results i) identify active LINE-1 retrotransposon families as the major source of functional RT in transformed cells, and ii) indicate that LINE-1-encoded RT might be regarded as a novel target in cancer differentiation therapy. Phase II trials are actually in progress to assess the therapeutic efficacy of EFV on metastatic prostate carcinoma patients (clinicaltrials.gov/ct2/show/NCT00964002?term=NCT00964002&rank=1). In contrast with the empirical efficacy of RT inhibitors, however, the molecular role of RT in cancer genomes is still elusive.

Here we report that treatment of A-375 melanoma cells with EFV causes a global reprogramming of the transcriptome, involving both coding and non-coding sequences with key roles in establishing cancer-repressive or -permissive conditions. We describe an active role of the endogenous RT in tumorigenesis, not only via transposition-associated integration events, but also by regulating the production of small non-coding regulatory RNAs.

## RESULTS

### RT-expressing cancer cell lines but not RT-deprived normal cells are sensitive to the RT inhibitor EFV

In past work we found in time-course assays that exposure of cancer cell lines to RT inhibitors reduced proliferation and promoted differentiation (substantiated by morphological and molecular changes) after 4-5 days of treatment [22; 23]; prolonged treatment maintained these features, whereas the cells quickly returned to their original conditions upon discontinuation of the treatment [23]. Prior to addressing the molecular mechanisms through which RT inhibitors regulate these changes, we first comparatively assayed the effect of RT-inhibitory treatment in human cancer cell lines of unrelated origin, i.e. A-375 melanoma, PC3 prostate carcinoma, U87 glioblastoma and Saos-2 osteosarcoma and, for comparison, in human non-transformed WI38 fibroblast cells, testing increasing concentrations of EFV.

**Figure 1:**
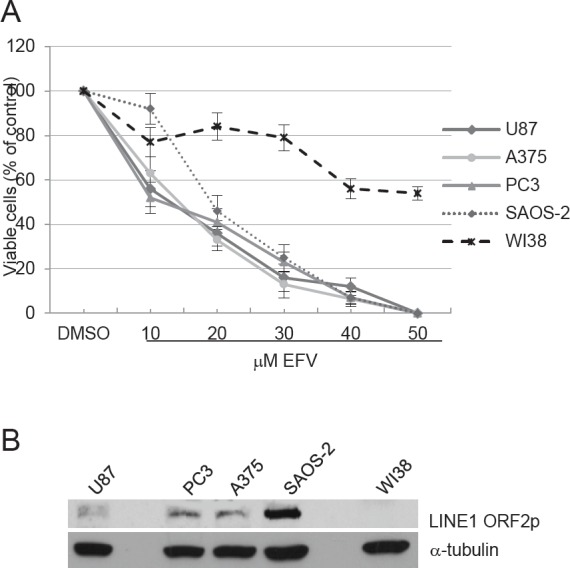
A) EFZ inhibits proliferation in human transformed cell lines Cells were cultured in the presence of increasing EFV concentrations for four days. The proliferation rate is expressed as the percentage of counted cells relative to the initial number of seeded cells, taken as 100. Histograms represent the mean value and bars the s.d. from at least three independent assays for all cell types. B) Western analysis of LINE-1 ORF2 protein (upper panel) and b-tubulin (lower panel) in whole cell extract (WCE) from the indicated cell lines.

The results summarized in Fig.1A show that low concentrations of EFV (10-20 uM) reduced the rate of proliferation of all four tumor cell lines by about 50% after 96 hours and higher concentrations (40-50 uM) fully abrogated it; in contrast, proliferation of normal WI38 was not, or only mildly, affected even with the highest concentration. These results confirm that the antiproliferative effects of EFV can be appreciated as early as 4 days after onset of the treatment in a variety of unrelated cancer-derived cell types, while undescoring a markedly reduced sensitivity of non cancer cells. It was relevant to assess the RT protein levels in the examined cell lines. LINE-1-encoded RT derives from the LINE-1 ORF2 product (ORF2p), a single 150 kDa polypeptide containing endonuclease (EN), reverse transcriptase (RT) and cysteine-rich (CYS) domains. We used a specific antibody directed against the LINE-1-encoded ORF2p in Western blot analysis of total proteins extracted from the cell lines analyzed in Fig. 1A. Results in Fig. 1B show that LINE-1-derived ORF2p, encoding the RT activity, is expressed in all cancer cell lines (most abundantly in Saos-2 cells) while being undetectable in normal WI38 fibroblasts. Together these results provide further support to the notion that the LINE-1-encoded RT is highly expressed in cancer but not in normal cells and confirm the selective sensitivity of cancer cells to RT inhibition by EFV.

### Alu- and LINE-1-containing hybrid RNA:DNA structures are present in transformed cells and are abrogated upon RT inhibitory treatment

LINE-1-encoded RT catalyzes the reverse transcription of both LINE-1 and Alu RNAs as a key step in the process of retrotransposition [29]. Both retrotransposon families act as sources of regulatory double-stranded RNA (dsRNAs) [30]. We wondered whether RT inhibition by EFV affected the production of dsRNAs in tumor cells. As newly reverse-transcribed cDNA copies would be virtually undistinguishable from the original DNA templates, we focused on the production of intermediate RNA:DNA hybrid products as a read-out of RT activity: we reasoned that such hybrid molecules might form preferentially in tumor cells, where LINE-1 encoded RT activity is higher compared to normal cells [21 and Fig. 1B]. If so, the formation of hybrid molecules might hinder the production of regulatory dsRNAs in cancer cells, but RT inhibitors should restore it. Such hybrids might also represent novel molecular markers in cancer cells.

**Figure 2:**
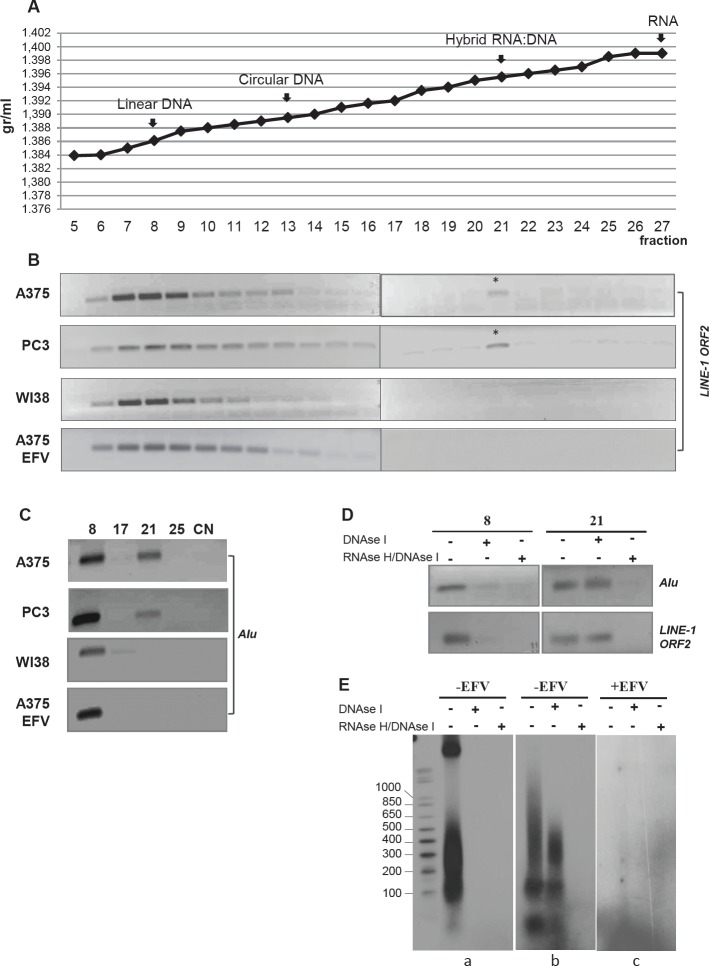
Identification of RNA:DNA hybrid structures through EtBr/CsCl density gradient centrifugation A) Buoyant densitiy of linear DNA, circular DNA, RNA:DNA hybrid and RNA used as density markers. Density values are indicated on the y axis and gradient fractions on the x axis. B) PCR amplification of LINE-1 sequences throughout the gradient fractions from untreated A-375, PC3 and WI38 cell lines and EFV-treated A-375 cultures. The asterisk marks LINE-1-specific amplification products in fraction 21. C) PCR amplification of Alu sequences throughout the gradient fractions as in B; only relevant fractions are shown. Numbers indicate the gradient fractions. NC, negative control. D) Alu and LINE-1 PCR amplification of aliquots withdrawn from fractions 8 and 21 of the A-375 DNA-containing gradient, treated with either DNase I alone or sequentially with DNase I + RNase H. E) Samples were labeled by random priming and analyzed through a 1.5% agarose gel. Panel (a) shows fraction 8 and (b) fraction 21 from untreated A-375 cell samples; panel c shows gradient fraction 21 from EFV-treated A-375 sample.

To address this issue, we compared DNA preparations from untreated A-375 melanoma, PC3 prostate carcinoma, non-transformed WI38 human fibroblasts and EFV-treated A-375 cells in EtBr-containing CsCl buoyant density centrifugation assays [31]. Linear and closed circular plasmid DNA, ^3^H-end labelled poly dA:U (DNA:RNA hybrid) and ^3^H-end labeled polyU (RNA) were used as density markers (Figure 2A). Gradient fractions were then analyzed by direct PCR amplification using LINE-1 ORF2- and Alu-specific oligonucleotide pairs.

LINE-1-containing sequences were identified in fractions 6 to 12, peaking in fraction 8, in samples from all cell types (Figure 2B): this corresponds to the density of linear DNA marker and indicates that the DNA bulk from all cell samples have comparable buoyant density. An additional LINE-1 amplification product was also identified in gradient regions of higher density, peaking at fraction 21 (starred in Figure 2B), which overlaps the buoyant density of the RNA:DNA hybrid marker. Hybrids were detected in native A-375 and PC3 tumor cell lines, but neither in non-transformed WI38 fibroblasts nor in EFV-treated A-375 cells.

The gradient fractions were analyzed by PCR using Alu-specific oligonucleotide pairs (panel C): we found Alu DNA amplification products peaking in fraction 8 in all four samples, and in fraction 21 only in A-375 and PC3 DNA samples, but neither in non-transformed WI38 nor in EFV-treated A-375 DNA.

To conclusively demonstrate that molecules in fractions 8 and 21 respectively contained double-stranded DNA (fraction 8) and RNA:DNA hybrid molecules (fraction 21), we processed them for differential enzymatic digestion, i.e. with either DNAse I only (cleaves single- and double-stranded DNA), or with a combination of RNAse H (cleaves the RNA component of RNA:DNA hybrids) and DNAse I, then analyzed the products by PCR using both LINE-1 and Alu oligonucleotide pairs. Indeed, DNAse I digestion abrogated both LINE-1- and Alu- amplification products from fraction 8, while not affecting the amplification products from fraction 21 (Figure 2D); the latter were abrogated after RNAse H and DNAse I double digestion. For further characterization, samples from fractions 8 and 21 were P^32^–labeled by random priming and fractionated on 1,7 % agarose gel to determine their size (Fig.2Ea): fraction 8 showed two labeled components, one of high molecular weight, corresponding to bulk DNA and another one of smaller size ranging from 500 to 100 bp. Both components contain normal DNA because they are fully degraded upon digestion with DNAse I alone. As expected, fraction 21 (Fig. 2Eb) lacks the high molecular weight component; the labeled material is distributed in a smearing pattern below 1 kb, with two discrete components of about 100-150 bp and 40 bp, respectively. Differently from fraction 8, most of the material recovered from fraction 21 was DNAse I-resistant, yet was fully degraded after combined DNAse I/RNase H digestion. This again confirms that fraction 21 mostly contains DNA:RNA hybrid molecules, the formation of which is fully abrogated when A-375 cells are pretreated with EFV (Fig. 2Ec). These results demonstrate that Alu- and LINE-1-containing hybrid RNA:DNA structures selectively form in transformed cell lines, which contain abundant RT protein, are absent from non transformed cells and are abrogated in transformed cells treated with RT inhibitor.

### RT inhibition changes the global transcriptome of A-375 melanoma cells

**Figure 3:**
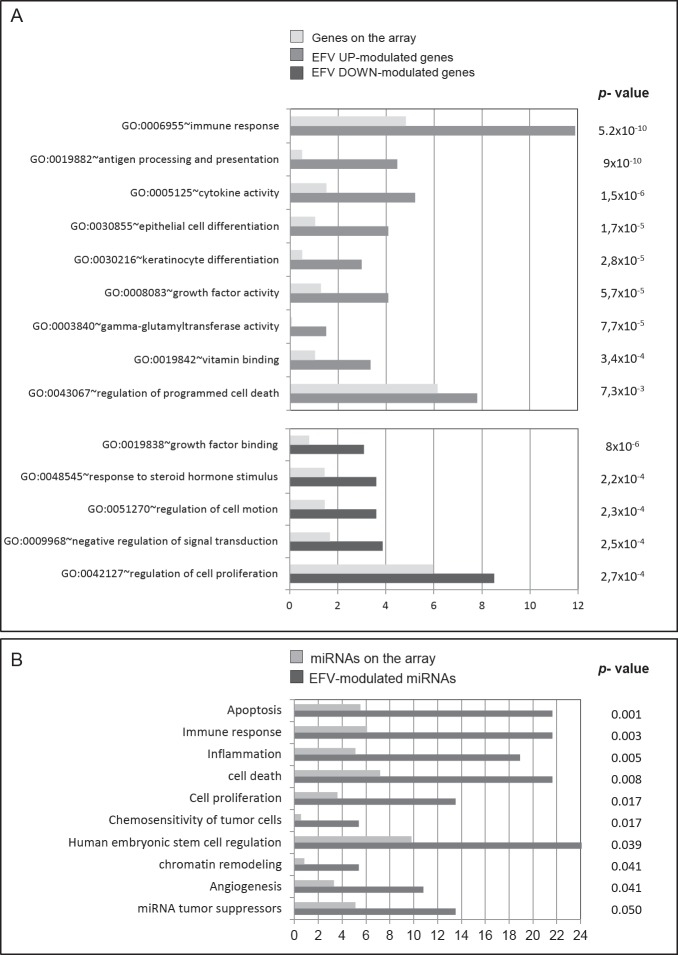
Gene ontology classification of EFV-modulated genes and miRNAs A) Gene Ontology classification of up- (gray histograms) and downregulated (dark histograms) genes in EFV-exposed A-375 cell cultures (at least 2.0-fold change, unpaired T-test P-value<0.05), using the David software. Biological process and Molecular function classes in which the genes fall are ranked according to the percentage of genes fitting each class relative to their expected frequency by chance (light gray histograms), calculated on the basis of the global array composition. The P value from the modified Fisher test classes enrichment (p < 0.05) is shown. B) Gene Ontology classification of differentially expressed miRNAs in EFV-exposed A-375 cell cultures (2.0-fold change, unpaired T-test P-value<0.05), using the Tool for annotations of human miRNAs (TAM, http://cmbi.bjmu.edu.cn/tam). The fold-enrichment values shows the overrepresentation of biological functions involving EFV-sensitive miRNAs (including up- and downregulated miRNAs), expressed as the pecentage of miRNAs fitting each class (gray histograms) relative to their expected frequency by chance, calculated on the basis of the global composition of the array (light gray histograms).

The findings in Fig. 2 that EFV inhibits DNA/RNA hybrid formation in cancer cells parallels previous results showing that pharmacological RT inhibitory treatment [22, 23], as well as RNAi to active LINE-1 elements [23, 26], corrected the transformed phenotype of transformed cell lines and concomitantly modulated the expression of specific differentiation and proliferation genes. To gain insight into the mechanism through which RT governs these phenomena, we decided to carry out a comprehensive analysis of RT-dependent changes in global RNA transcription. A-375 melanoma cells were treated with 20 uM EFV (or DMSO for control), a concentration that avoids massive cell death yet has cytostatic effect on cancer cells (Figure 1 and ref. 21). RNA was extracted and subjected to microarray analysis (details in materials and methods) focusing on: i) protein-coding genes, ii) miRNAs and iii) ultraconserved regions (UCRs), a class of genomic sequences frequently located at chromosomal fragile sites and originating a subset of non-coding RNAs whose expression is altered in human cancers [32-34]. The results indicate an extensive reprogramming of transcription profiles for all three classes of sequences in EFV-treated compared to untreated A-375 cells. 854 coding genes were found to be modulated by EFV, 456 of which were down- and 398 upregulated (full list in supplementary Table S1, Moderated T test, corrected p-value≤0.05, fold change ≥2). Supplementary Figure S1 shows a qPCR validation for four of them. Several EFV-downregulated genes are markers of cancer and metastatic growth (e.g., MYCN and BMX, both overexpressed in a variety of tumors); conversely, the upregulated group includes genes with growth suppressive activity, e.g. ANGPTL4 (prevents metastatic progression by inhibiting vascular activity, tumor cell motility and invasiveness), RASAL1 (suppresses cell proliferation and transformation ability of gastric cancer cells) and Rap1GAP (a negative regulator of cancer cell proliferation and invasiveness). The Gene Ontology classification of EFV-modulated genes (Figure 3A), analyzed using the Fisher Modified Exact (p-value ≤0.01), revealed that EFV-downregulated genes are enriched in the classes of growth factor binding, cell proliferation, signal transduction cell motion and response to steroid hormones, while upregulated genes show, among others, a significant enrichment in genes related to the immune response, pathways of cell differentiation and growth factor activity.

We next examined miRNA expression profiles in control and EFV-treated A-375 cells, and found that 35 out of 726 analyzed miRNAs showed significant variations after EFV treatment (list in supplementary Table S3; T test corrected p-value < 0.05, fold change ≥1.5); of those, 19 were down- and 16 upregulated. The functional annotation analysis of the resulting miRNA list, performed using the TAM online tool, depicted a significant enrichment (p-value <0.05) in cell proliferation-, tumor chemosensitivity-, chromatin remodeling-, apoptosis-, cell death and tumor suppression-associated miRNAs (Figure 3B). The same analysis conducted to detect any possible overrepresented miRNA clusters showed that about one third of the downregulated miRNAs belong to the hsa-mir-1283 cluster (data not shown), a primate-specific gene cluster characterized by a high enrichment in flanking Alu sequences, thought to have co-evolved with the miR cluster [35].

We noticed that most EFV-modulated miRNAs are directed towards oncogenes or tumor suppressors and typically localize close to or at cancer-associated genomic regions (CAGRs) and fragile sites (Table 1). Remarkably, ten EFV-modulated miRNAs classify as metastamiRs (Table 2), a miRNA subpopulation with crucial roles in tumor progression, invasiveness and metastasis [reviewed in 34]. EFV treatment reversed their original expression profiles in A-375 melanoma cells (Table 2) and specifically upregulated those that are underexpressed in cancer while downregulating the overexpressed ones, with the exception of miR-125b, which can be either under- or over-expressed in different tumors [37].

**Table 1:** EFV-modulated miRNAs implicated in tumor progression and localized at cancer associated genomic regions (CAGR) and fragile sites (FRA)

Name	EFV modulation	Preferential sites of expression	Chr	CAGR localization	References
miR-16a	down	CLL, ALL, oral ca	13	D13q14-14.3	56
miR-21	down	CLL	17	FRA17B	56
miR-25	down	HCC	7	FRA7F	33
miR-32	down	prostate ca, HML	9	FRA9E	56, 33
miR-33a	down	CRC, astrocytoma	22	D 22q12.2-q13.33	56
miR-132	down	HCC	17	D 17p13.3	56
miR-181a-2	down	head and neck ca	9	D 9q33-34.1	56
miR-181c	down	osteosarcoma	9		57
miR-23b	up	CLL	9	FRA9D	56
miR-34b	up	breast ca	9		59
miR-125b-2	up	lung ca, myeloid and lymphoid leukemia, breast ca, glyoblastoma	21	D 21q11.1	56
miR-146a	up	CLL	5		58
miR-148a	up	ovarian ca	12		60
miR-193	up	ovarian ca	17	D 17q11.1	56
miR-199	up	bladder ca	9	D 9q33-34.1	56
miR-513a-5p	up	male infertility	X	FRAXA, RAXE	33

CLL, chronic lymphocytic leukemia; HCC, hepatocellular carcinoma; ALL, adult lymphoblastic leukemia; *HML, human* myeloid *leukemia*; CRC, colorectal carcinoma

**Table 2:** EFV-modulated metastamiRs, miRNAs promoting tumor progression, invasiveness and metastasis

Name	EFV modulation	Modulation in cancer	Biological correlation	Cancer type	References
miR-21	down	up	Correlated with invasion and metastasis	lung, colorectal,	61,62
miR-33a	down	up	Dysregulated expression in bone metastasis from primary prostate	prostate	63
miR-181a	down	up	Related with shortened disease-free survival, highly upregulated in osteosarcoma	osteosarcoma	57
miR-199b	down	up	Expression dysregulated in metastasis	brain	64
miR-34b	up	down	Downregulated in metastasis, reactivated upon drug treatment inhibits tumor growth and lymph node metastasis	colorectal, melanoma, head and neck	65
miR-125b	up	down/up	Downregulated in breast and upregulated in i cancer, association with cancer metastasis	breast, colorectal	37
miR-146a	up	down	Inversely correlated expression with cancer progression and metastasis	prostate, breast	66
miR-148a	up	down	Downregulated in metastasis, acts as metastasis suppressor inhibiting tumor growth and lymph node metastasis	colorectal, melanoma, head and neck	65
miR-193b	up	down	Inversely correlated expression with cancer progression, invasion and metastasis	breast	67
miR-204	up	down	Highly reduced expression in cancer progression; overexpression suppresses invasiveness and acts as metastasis suppressor	head and neck	68

We also examined UCRs (481 sequences) [38] and found that EFV significantly modifies the expression of 52 of them (p-value < 0.05, list in supplementary Table S3, features are illustrated in Table 3). An analysis of the genomic organization of EFV-modulated UCRs revealed that 25 are upregulated and comprise mostly (52%) non-exonic (N) elements, followed by 40% of possibly exonic (P) and only 8% of exonic (E) elements. In contrast, among 27 down-regulated UCRs, 78% are exonic, whereas non-exonic and possibly exonic elements account for only 15 and 7%, respectively. As in the case of miRNAs, a large proportion of both up- and downregulated elements are involved in human cancers and/or structurally associated with loss of heterozygosity (LOH) and fragile sites (Table 3). In synthesis, therefore, the RT inhibitory treatment causes global changes in the transcriptome of melanoma cells, affecting both coding and non-coding sequences, many of which are involved in cancer onset and progression.

**Table 3:** EFV-modulated UCRs implicated in tumor progression and localized at cancer associated genomic break sites

Name	EFV modulation	Type	Length	Chr	Break sites	Tumor	References
uc.24	down	n	336	1	LOH		32
uc.28	down	e	355	1	FRA		32
uc.135	down	e	201	3	EVI1	CLL	58
uc.144	down	e	205	4		CLL/CRC	58
uc.193	down	e	319	6	LOH		32
uc.194	down	e	201	6	LOH		32
uc.215	down	n	262	7			32
uc.276	down	p	432	9	LOH		32
uc.308	down	p	277	10	LOH		32
uc.343	down	e	388	12	FRA12A/LOH		33, 32
uc.345	down	e	301	12	FRA12A		33
uc.388	down	n	298	15	High CRC	CRC	32
uc.457	down	e	211	22		CRC	
uc.20	up	p	269	1		HCC	
uc.38	up	n	224	1	FRA1F		33
uc.158	up	n	224	5		CRC	
uc.234	up	p	272	7		CRC	
uc.274	up	p	327	9	LOH	HCC	32
uc.292	up	e	217	10	MLR2	CRC	32
uc.295	up	n	209	10	LOH		32
uc.303	up	n	272	10	LOH		32
uc.352	up	n	200	13	FRA	CLL	32
uc.365	up	n	278	14	LOH		32
uc.398	up	p	322	16		CRC	

FRA, fragile site; LOH, loss of heterozygosity; CRC, colorectal ca; CLL, chronic lymphocytic leukemia; HCC, hepatocellular carcinoma.

### EFV-responsive miRNAs, UCRs and protein-coding genes are significantly enriched in Alu or LINE-1 retrotransposons in their reference sequence

The finding that exposure to EFV modulates the transcriptional profile of protein-coding-, micro- and UCR- subpopulations raised the question of whether these sequences might be physically associated to retrotransposons. To address this question we analyzed the EFV-modulated sequences using the RepeatMasker program to annotate any transposable elements either within, or flanking (+/− 100 Kbp), members in each of these classes (details in Materials and Methods). EFV-insensitive members from the same class were used as negative controls (see full list in supplementary Tables S4, S5, S6).

**Figure 4:**
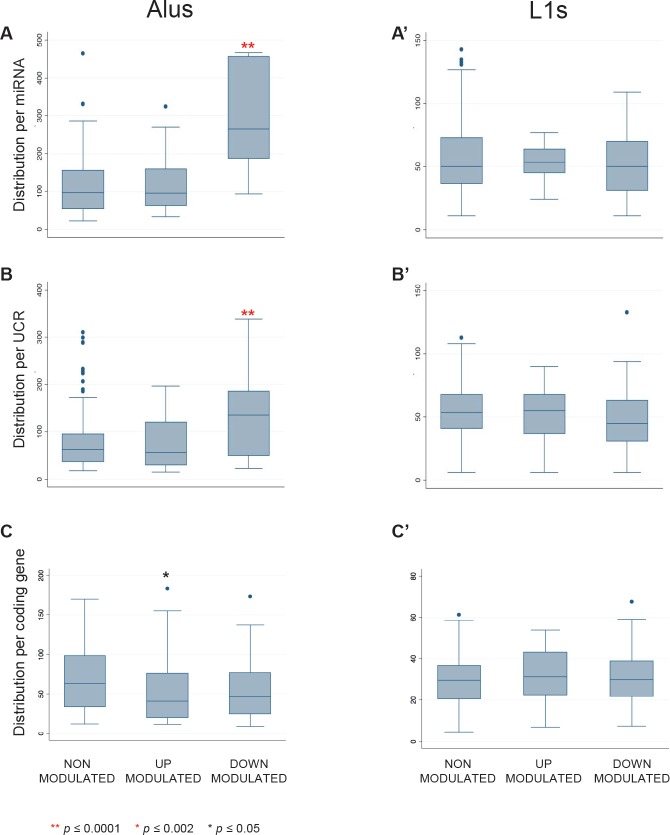
Alu and LINE-1 content around miRNAs, UCRs and coding genes The box and whisker plots represent the distribution of numbers of repeated Alu (left panels) and LINE-1 (right panels) elements flanking (+/− 100 Kbp) the indicated sequence classes, i.e. miRNAs (A and A'), UCRs (B and B') and protein-coding genes (C and C'). EFV-downregulated miRNAs (A) and UCRs (B) are highly significantly enriched (**, P≤0.0001) in Alu elements.

Significant associations were found between EFV-sensitive sequences and retrotransposons. EFV-modulated miRNAs (P <0.0001, supplemetary Figure S2B) and UCRs (P=0.0003, supplemetary Figure S2C) showed statistically significant over-representation of flanking Alu elements compared to EFV-insensitive sequences. In particular, a significant enrichment in Alu content was found in EFV-downregulated subpopulations of miRNAs (Figure 4A) and UCRs (Figure 4B), whereas upregulated sequences did not significantly differ from non-modulated controls. No significant variations were found instead in the distribution of LINE-1 elements among the three groups of sequences (Figure 4A', B', C').

### EFV-downregulated miRNAs and UCRs are significantly enriched in Alu pairs arranged in inverted orientations (IRAlus)

**Figure 5:**
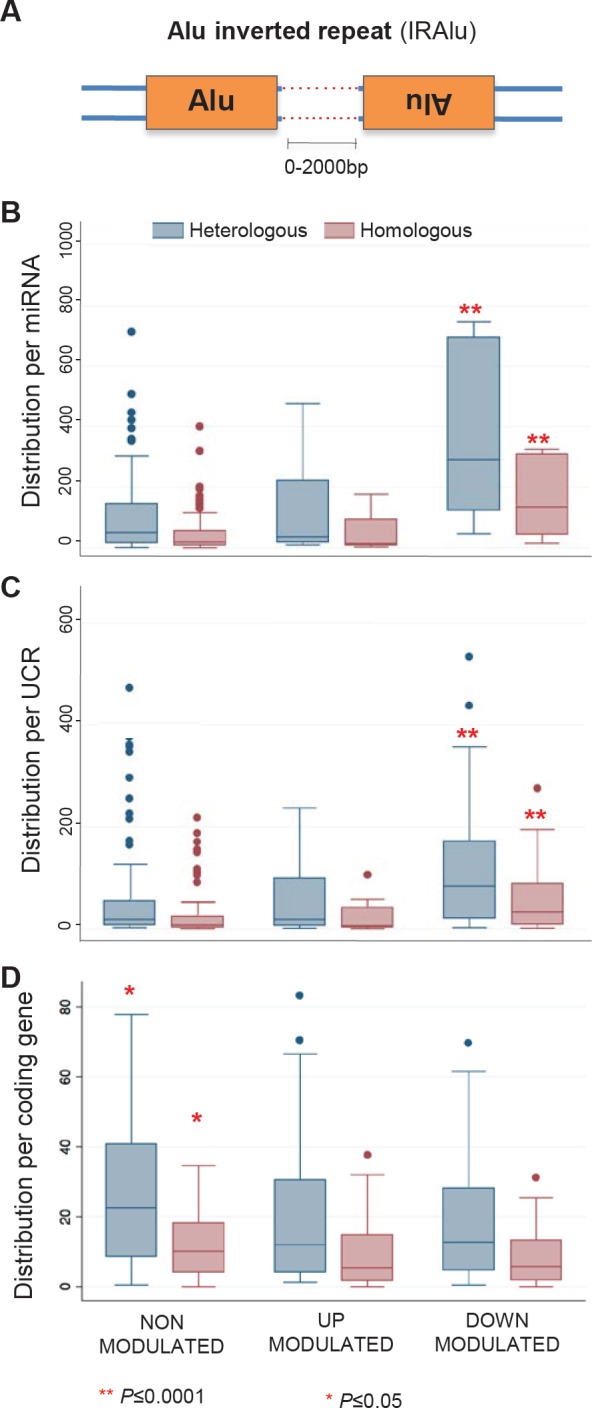
Inverted Alu repeats (IRAlus) distribution around miRNAs, UCRs and coding genes IRAlus in which inverted repeats are spaced by less than 2.000 bp are considered (A). The box and whisker plots represent the distribution of numbers of IRAlus flanking (+/− 100 Kbp) the indicated sequence classes. IRAlus are highly significantly more abundant (**, P≤0.0001) among EFV-downregulated miRNAs (B) and UCRs (C), but not among EFV-modulated coding genes (D). Blue box plots represent heterologous IRAlus (i.e., Alu inverted repeats from two distinct subfamilies), red box plots represent homologous IRAlus (i.e., both Alu repeats from the same subfamily).

Closer inspection of EFV downregulated miRNA and UCR populations revealed that a large proportion of their Alu content is made up of pairs of typical inverted Alu repeats (IRAlu), in which Alu elements are arranged in opposite orientations (Figure 5A). We annotated IRAlus in which the two sense/antisense arranged Alu elements were separated by less than 2.000 base pairs (Figure 5A). For both miRNAs (Figure 5B) and UCRs (Figure 5C), IRAlu were strikingly more abundant among EFV-downregulated compared to upregulated or non-modulated sequence groups. In contrast, IRAlus were not significantly enriched among EFV-downregulated protein-coding genes (Figure 5D), in which IRAlus were similarly distributed among all subgroups.

The enrichment among EFV-downregulated miRNAs and UCRs was confirmed when considering only homologous IRAlus (red histograms in Figure 5), in which both Alu repeats belong to the same subfamily; this is interesting, as 82% of all homologous IRAlus belong to AluS elements, one of the most ancient Alu subfamilies. IRAlus constitute 87.4% of all Alu sequences found in the vicinity of EFV-downregulated miRNAs, compared to only 72% of Alus near non-modulated or upregulated sequences (data not shown).

## DISCUSSION

Here we have combined microarray profiling, genomic bioinformatics analysis and nucleic acid analysis from density gradient fractions to investigate the molecular mechanisms through which the cellular endogenous RT, the major source of which is in active LINE-1 transposable elements, and required for retrotransposition of LINE-1 themselves and of non-autonomous Alu elements, influences cancer cell proliferation. We report that: i) exposure to the RT inhibitor EFV strongly inhibits proliferation of RT actively expressing cancer cell lines, while minimally affecting non transformed RT-deprived cells; ii) RNA:DNA hybrid structures, containing both Alu and LINE-1 sequences, are present in transformed but not in normal cells; iii) EFV treatment of transformed cells abrogates the formation of RNA:DNA hybrid structures; concomitantly, iv) EFV treatment modulates the expression of cancer-relevant miRNAs and coding genes in a genome-wide analysis of melanoma cells; v) EFV-downregulated sub-populations of miRNAs are significantly enriched in Alus, and particularly in pairs of inverted Alu repeats, IRAlu. These findings point to a central role of RT, as detailed in the model explained below.

### A proposed model for RT-mediated reprogramming of cancer transcriptome

**Figure 6:**
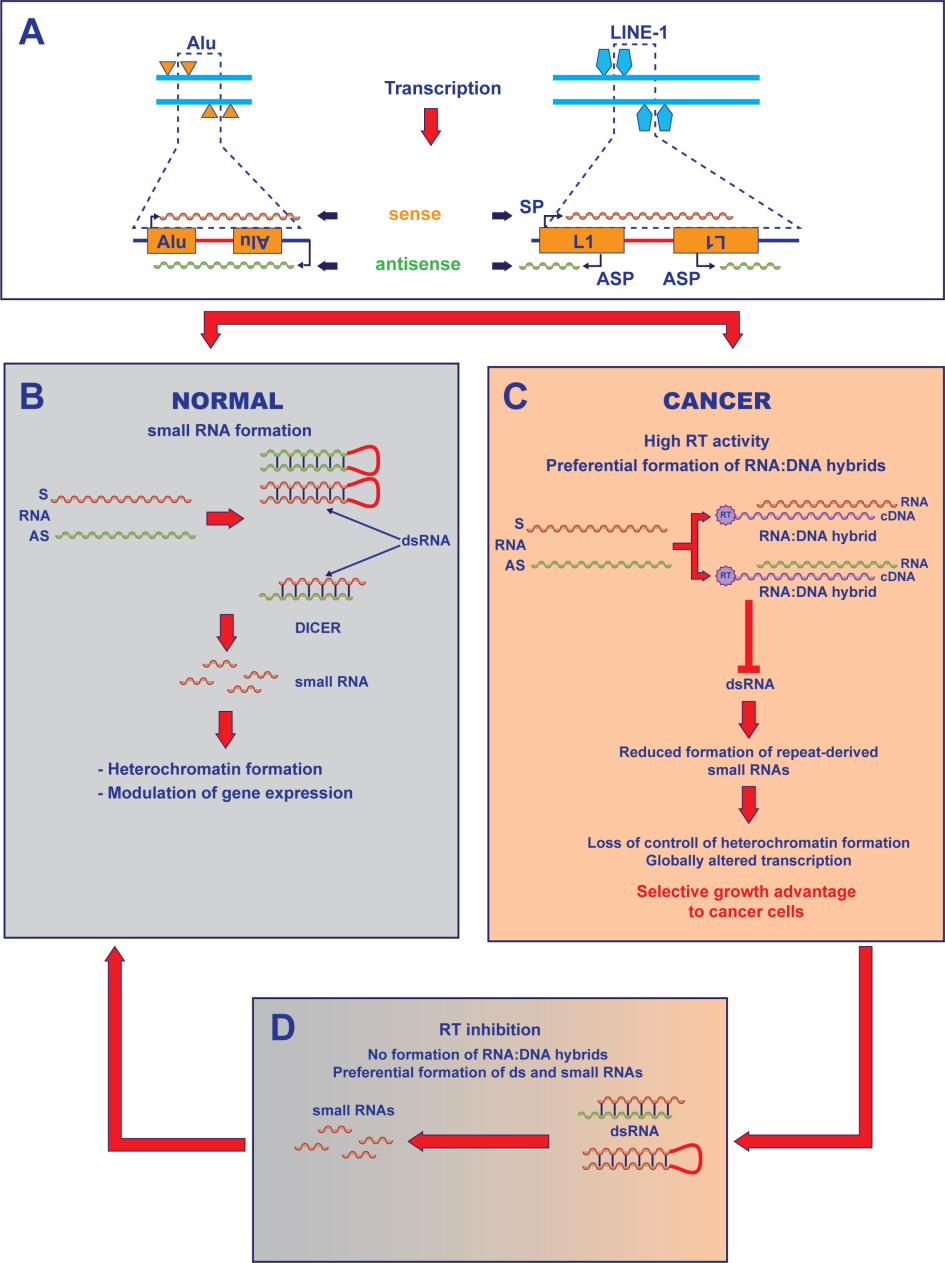
Model for RT-mediated control of the transcriptome in cancer cells A) Retroelements are distributed in the genome in tandem and inverted repeats (i.e. closely spaced elements with opposite orientation). The top left panel depicts four Alu elements in opposite orientation on complementary DNA strands. Below, RNA transcripts (sense, red; antisense, green) containing two Alu retroelements in opposite orientation can be generated either by the internal Alu promoters, or from one Alu promoter and a nearby gene promoter in antisense orientation (external black arrows) provided by the host genome. LINE-1 elements (right) are endowed with sense (SP) and antisense (ASP) promoters, supporting LINE-1 sense and antisense transcription. B) In normal cells, double-stranded RNA (dsRNA) forms either via intramolecular base paring of an RNA containing two oppositely orientated retroelements, or via base pairing between sense (S) RNA and antisense (AS) RNAs. dsRNAs are processed by Dicer into small regulatory RNAs. C) In cancer cells, the RT encoded by highly active LINE-1 elements reverse-transcribes the available RNA populations: RNA:cDNA hybrid molecules form efficiently (Figure 3B) to the detriment of dsRNA. D) EFV treatment of cancer cells inhibits the RT activity, reduces the formation of RNA:cDNA hybrid and restores the control of regulatory small RNAs, causing the epigenetic conversion to a normal phenotype.

The present findings suggest a model for the role of LINE-1-encoded RT in the transition from normal to tumorigenic transcriptome (Figure 6). The model builds on the evidence that, in normal cells, LINE-1 and Alu retrotransposons can generate dsRNAs [30] through several pathways: intramolecular base paring of mRNAs containing two oppositely oriented retroelements [9,39], or base pairing between sense (S) RNA and antisense (AS) transcripts - the latter originating from antisense promoters occasionally provided by the host genome at nearby loci [40] - or, for full-length LINE-1 elements, via transcription from sense (SP) and antisense (ASP) promoters located in their 5'-UTRs [12] (Fig. 6A). Retroelement-derived dsRNAs are cleaved by Dicer into small RNAs that have roles in regulation of the transcriptome [41] and in heterochromatin organization [42] (Fig. 6B).

LINE-1 are overexpressed in cancer cells and RT activity is correspondingly high [reviewed in 6; also Fig. 1 in this paper]. In these cells, the abundant RT can intercept and reverse-transcribe Alu and LINE-1 RNA molecules (Fig. 6C), as indicated here by the presence of DNA:RNA hybrids exclusively in cancer cells (Fig. 2). Such hybrid molecules have a potential to impair the formation of dsRNAs. We find that DNA:RNA hybrid structures do not form in EFV-treated melanoma cells (Fig. 2B, C, Ea), suggesting that the RT inhibitor has indeed removed a major hurdle in the biosynthesis of Alu-derived small RNAs, thereby reestablishing the physiological supply of regulatory small RNAs typical of cancer-restrictive conditions. In other words, the model in Fig. 6 highlights a previously unrecognized role of LINE-1-encoded RT in disrupting miRNA-based regulatory mechanisms operating in normal cells. Retrotransposon-derived DNA:RNA hybrid molecules are found in cancer cells of unrelated origin, suggesting that they are a common feature in different cancer types. Interestingly, we have identified discrete amounts of DNA:RNA hybrid molecules in colon adenocarcinoma bioptic samples; only faint traces were present in the neighboring colon tissue from the same patient, deriving perhaps from infiltrating cancer cells in the non-transformed tissue surrounding the tumor (unpublished results). These data, to be extended in further studies, confirm that RNA:DNA hybrids form in primary cancer tissues.

The distinctive presence of the hybrid molecules in cancer cells is consistent with the general observation that both LINE-1-derived siRNA [43] and miRNAs [44] are globally downregulated in tumor compared with normal cells. Here we have used A375 melanoma cell cultures to explore in depth the significance of the DNA:RNA hybrids. We find that EFV-mediated RT inhibition, parallel to preventing the formation of DNA:RNA hybrids in A375 cells, induces an extensive remodulation of the global transcriptome, an ultimate determinant of the cell fate (supplementary Tables S1, S2, S3), involving coding genes, miRNAs and UCRs implicated in proliferation, signaling and growth fractor pathways. We propose that RT inhibition suppresses the “cancer-promoting” conditions that had lead to defective differentiation (or dedifferentiation) and active proliferation, and restores a “cancer-repressive” state.

### The link between retrotransposons and small RNAs: functional implications

The RT-dependent mechanism described here, and its origin in active LINE-1 elements, strengthen the emerging links between the retrotransposon machinery and small RNA-based regulatory mechanism(s) [35, 39, 45]. A high proportion of small RNAs originated and evolved from retrotransposon families [39], e.g. MIR/LINE-2 [45, 46], Alu [47, 35, 39, 9], LINE-1 [12, 44, 39] and LTR-containing [44, 39]. Alu-specific dsRNAs are generated in normal cells via intramolecular base paring of mRNA containing two oppositely oriented retroelements. The present data indicate that LINE-1-encoded RT can disrupt small RNA-mediated regulatory mechanisms, a well-documented occurrence in cancer [48]. Our finding that EFV preferentially downregulates sequences enriched in Alus and IRAlus is also consistent with this idea.

The model in Fig. 6 proposes a key role for the endogenous RT, and hence for LINE-1 and Alu repetitive elements that depend on it, on the global production of miRNAs, as part of a functional network that ultimately targets the expression of protein-coding genes. The network has a genome-wide reach, including ncRNA-coding sequences such as UCRs which, consistently, are also modulated upon RT inhibition (Table 3, supplementary Table S3). UCR expression is regulated by direct interaction with miRNA [32,34]: while accounting for their sensitivity to RT inhibition as observed in our experiments, this would place UCRs downstream of the RT-dependent regulatory circuit identified here. The proposed model can also accommodate a role for long ncRNAs, which are increasingly being identified as key epigenetic players in various regulatory activities, two thirds of which contain retroelement sequences [49 and references herein].

Pseudogene-derived RNA transcripts have been recently hypothesized to serve as “perfect decoys” for absorbing specific miRNAs that otherwise affect the expression of the ancestral protein-coding genes. This hypothesis, referred to as the ceRNA hypothesis (competing endogenous RNA), views the cross-talk between reverse-transcribed cDNA copies and miRNAs as the basis for the establishment of “large-scale regulatory networks across the transcriptome” [50]. The RT-dependent mechanism in Fig. 6 presents a distinct yet conceptually compatible scenario, in that the non-coding component of the genome emerges as key not only to shaping the global transcriptome, but also to balancing the output of productive miRNAs through regulatory cross-talks.

In preliminary assays, we have found that EFV modulates epigenetic marks and promotes a global reorganization of nuclear chromatin, with a significant increase in both heterochromatic foci and in histone H3 K9 methylation (data not shown). These findings highlight the nature of the LINE-1-encoded RT as an epigenetic regulator and is consistent, in retrospect, with earlier findings that RT repression (via RT inhibitors) and reactivation (following discontinuation of the inhibitory treatment) reversibly shift the cell status from normal to tumorigenic [22, 23, 26]. This conclusion is in agreeement with the view that genetic and epigenetic mechanisms are not necessarily separate events in oncogenesis [51].

A tempting parallel emerges between the impairment, or partial inactivation, of small RNA-dependent regulatory mechanisms associated with high levels of LINE-1-encoded RT observed in cancer cells and the global suppression of miRNA function occurring during mouse preimplantation development [53], in stages in which the RT-dependent mechanism is also highly active [53,16]. This analogy suggests that the loss of small RNA function is a general consequence of the activation of the RT-dependent mechanism, a feature observed in both tumor cells and embryos.

## CONCLUSIONS

The present results extend previous findings that LINE-1 elements, and their RT product, are expressed at low levels in normal cells and abundantly in transformed cells, and that RT inhibitory treatments restore differentiation and reduce cell proliferation in transformed cells. Moving one step further, the experiments reported here identify an unsuspected role of RT in regulating the production of small RNAs implicated in these events. In normal cells, small regulatory RNAs are generated from retroelements, a well-estabished source of double-stranded RNA (dsRNAs) production. Our present findings indicate that, in transformed cells, the endogenous RT reverse-transcribes retroelement-derived transcripts and generates DNA:RNA hybrid structures. We propose that this impairs the production of dsRNAs that constitute the normal substrate for Dicer cleavage, altering the formation of regulatory small RNAs, with a direct impact on the global transcriptome. RT inhibition restores the small RNA-mediated mechanism of cellular control. These results disclose a novel LINE-1 RT-dependent mechanism that exerts a crucial role on proliferation and differentiation in transformed cells and strengthen the emerging view that retrotranposable elements do not only affect genome function via retrotransposition-dependent integration, but also via genome-wide epigenetic mechanisms regulated by the genes harbored within these elements.

## METHODS

### Cell culture and RT inhibition

Cell lines were cultured at 37°C in a 5% CO2 incubator. The human cancer-derived cell lines A-375 (melanoma), U87 (glioblastoma), Saos-2 (osteosarcoma) were cultured in DMEM; the PC3 (prostate carcinoma) cell line was cultured in RPMI 1640. The non transformed WI38 human fibroblast cell line (ATCC-CCL75) was cultured in Eagle's Minimum Essential Medium supplemented with 1% non essential aminoacids. All media were supplemented with 10% FBS, 1% L-Glutamine and 1% penicillin/streptomycin. In proliferation assays 5×10^4^ cells per well were seeded in mutiwell culture dishes. EFV was purified from commercially available Sustiva (Bristol-Myers Squibb) as described [22], dissolved in DMSO and added to the culture medium in the indicated concentrations and times.

### RNA extraction and array profiling of protein-coding genes, microRNAs and UCRs

For gene microarray and miRNA/UCR microarray analyses, RNA was extracted from A-375 cells treated for 12 days with 20 uM EFV or DMSO alone (controls, CTR) using the PureLINK RNA mini kit (Ambion) (gene microarrays) or Trizol (Ambion) (miRNA/UCR microarray). Three biological replicates were used for each microarray analysis.

### Microarray hybridization

miRNA expression was investigated using the Agilent Human miRNA microarray v.2 (#G4470B, Agilent Technologies), as detailed in [54]. Protein-coding gene expression was detected using the Agilent whole human genome oligo microarray (#G2509F, Agilent Technologies). Labeled cRNA was synthesized from 500 ng of total RNA using the Low Imput Quick-Amp Labeling Kit, one color (Agilent Technologies) in the presence of cyanine 3-CTP. Hybridizations were performed at 65°C for 17 h in a rotating oven. Images (5 um resolution) were generated by Agilent scanner and the Feature Extraction 10.7.3.1 software (Agilent Technologies) was used to obtain the microarray raw data.

A custom 8×15K UCR-specific array was developed using Agilent eArray (https://earray.chem.agilent.com/earray/) using the information derived from [38]. The array design is submitted to ArrayExpress database (accession number A-MEXP-2317). Hybridazions were performed according to the Agilent one-color gene-expression protocol, with the modification that T7_(N)6 random primers were used instead of the oligo (d)T-T7 primer.

### Microarray analysis

Microarray results were analyzed by using the GeneSpring GX 12.5 software (Agilent Technologies). Data transformation was applied to set all the negative raw values at 1.0, followed by a Quantile normalization and a log2 transformation. Filters on gene expression were used to keep only the miRNAs expressed (Detected) in at least one sample. Differentially expressed miRNAs were identified by comparing EFV vs. CTR samples. A 1.5 fold-change filter (for microRNAs and UCRs) or a 2 fold-change filter (for gene expression) followed by a moderated t-test, with Benjamini-Hochberg correction, were applied. The raw and normalized microarray data are submitted to ArrayExpress database and can be retreived using the following accession numbers: E-MTAB-1737 (microRNA), E-MTAB-1735 (gene expression), E-MTAB-1736 (UCR expression). Gene annotations were mined using web-based tools (DAVID, http://david.abcc.ncifcrf.gov, GeneCards, http://www.genecards.org/index.shtml). A modified Fisher Exact test was used for gene-enrichment analysis on Gene Ontology classification (by DAVID, http://david.abcc.ncifcrf.gov) using the composition of the Agilent Human miRNA Microarray (V2) as a background. Gene ontology classification of modulated miRNAs was performed using the TAM tool for annotations of human miRNAs (http://cmbi.bjmu.edu.cn/tam).

### Western immunoblotting

A-375, PC3, U87,SAOS-2 and WI38 cell cultures were lysed in RIPA buffer (50 mM Tris-HCl (pH 7.4), 150 mM NaCl, 1 mM EDTA, 1 mM EGTA, 1% (v/v) NP-40, 0.25% (w/v) deoxycholic acid) and 1x complete EDTA-free protease inhibitor cocktail (Roche Molecular Biochemicals). 60 µg of total proteins were fractionated through a 7.5% Mini-PROTEAN TGX Precast Gel (BIORAD 456-10237) and transferred using a Trans-Blot Turbo Transfer System (BIORAD 170-4155) on a nitrocellulose membrane (Mini Nitrocellulose Transfer Packs, (BIORAD 170-4158). Primary antibodies used were LINE1 (H-110, raised against amino acids 1081-1190, mapping near the C-terminus of human LINE-1 protein product) (Santa Cruz Biotechnology) and a-tubulin (Sigma-Aldrich). HRP-conjugated goat anti-rabbit IgG (BIORAD 170-6515) and goat anti-mouse IgG (BIORAD 170-6516) were used as secondary antibodies.

### EtBr-CsCl buoyant density gradients and analysis of gradient fractions

Genomic DNA was extracted from WI38, PC3 and A-375 cell lines in lysis buffer (50 mM TRIS-HCl pH 7, 2 mM EDTA pH 8, 1% SDS) supplemented with 50 ug/ml Proteinase K (Sigma-Aldrich) and 145 ug/ml RNAse A (Sigma-Aldrich) overnight at 37°C, followed by several phenol/chlorophorm extractions. Solid CsCl was added to a final concentration of 1.01 g/ml to 4 ml aliquots containing 20 ug DNA and 200 ug/ml of ethidium bromide [29] in polyallomer tubes (Beckman 342412) and centrifuged at 60,000 rpm for 24 h at room temperature in a VTi65 rotor in a Thermo Scientific WX Ultra 100 ultracentrifuge. 27 fractions (190 ul each) were collected, starting from the top, and their density was assessed in a refractometer. DNA was ethanol-precipitated from each fraction, washed in 70% ethanol, resuspended in 300 ul H2O and 5 ul aliquots from each fraction were PCR amplified using the indicated oligonucleotide pairs:

ORF2: Fw- TCCAGCAGCACATCAAAAAG; Rev- CCAGTTTTTGCCCATTCAGT
Alu115: Fw- CCTGAGGTCAGGAGTTCGAG; Rev- CCCGAGTAGCTGGGATTACA
GAPDH: Fw- GAGTCAACGGATTTGGTCGT; Rev- TGACAAAGTGGTCGTTGAGG

Where indicated, fractions 8 and 21 collected from the gradient were digested with either DNAse I alone, or simultaneously with RNAseH/DNAse I and subjected to PCR amplification. Circular and Bam H1-linearized pCMV-EGFP plasmid DNA (Clontech) were used as DNA density markers while [^3^H] end-labelled polyU (100 nt) and [^3^H] end-labelled poly dA:U were RNA and DNA:RNA hybrid density markers, respectively; densities of these latters were determined by mixing aliquots of gradient fractions with scintillation cocktail (Pico-Fluor 40, Perkin Elmer) and counting in a Beckman scintillation counter. To determine the product sizes, fractions 8 (bulk) and 21 (hybrid) from control and EFV-treated A375 cells were digested with DNAse I or RNAse H/ DNAse I, labelled with alpha-^32^P dCTP using the Invitrogen Random Primers DNA Labeling System kit (18187-013) and fractionated through 1.7% agarose gels; the gels were then dried and exposed to autoradiographic films.

### Retroelement enrichment analysis

UCR, miRNA and protein-coding gene sequences were classified in two groups: modulated elements (fold change, FC, >2 for genes, >1.5 for miRNAs and UCRs; P value<0.05 for all) and non-modulated elements. Using RepeatMasker [Smit, Hubley and Green. RepeatMasker Open-3.0 1996-2010, www.repeatmasker.org], we first annotated from the human Reference sequence all REs flanking (+/− 100 Kbp) each individual sequence of the three analyzed classes (see supplementary Tables S5, S6, S7). Gene-related elements were computed as “element density”, i.e. element number divided by gene length, then multiplied by 100.000; this procedure enabled a direct comparison of the retroelement number among genes of different length. We then compared the number of retroelements from each family in EFV-modulated genes, miRNAs and UCRs (modulated group) with matched non-modulated members of the same classes (see supplementary Figure S2 and Figure 4). Statistical analyses were carried out using STATA. The RE content was marked for each member of the analyzed classes (genes, miRNAs, UCRs); a one-way ANOVA test was then performed for each RE family between the modulated and non-modulated group.

### Real-time PCR

Quantitative PCR was performed using a 7300 Real-Time PCR System (Applied Biosystems) and SYBR™ Green JumpStart™ Taq ReadyMix™ (Sigma-Aldrich Co). 50 ng of cDNA from EFV-treated and control (DMSO-treated) (4 days) A-375 cells were used. The relative amounts of transcript were analyzed for *serpin-1*, tachykinin (precursor 1), *sox-11* and *hmox1* genes using the 2-DDCt method [55] and normalized to *beta-actin*. The following primers were designed using the Primer Express™ software v2.0 (Applied Biosystems):

*serp1*: Fw-CCCGGATCGTCTTTGAGAAG; Rev- TCCAGAGGTGCCACAAAGCT
*tac1*: Fw-AATTACTGGTCCGACTGGTACGA; Rev- AAAGGGCTCCGGCAGTTC
*sox-11*: FW-ACATGGTATTCTTGCCACTGGA; Rev- CCAAAATGCCATCAGAGTCTGT
*b-actin*: Fw-GCCGGGACCTGACTGACTA; Rev- TGGTGATGACCTGGCCGT
*hmox1* was analyzed using the PrimePCR™ SYBR™ Green Assay (BIORAD cat. 100-25636)

## Supplementary Figures and Tables





## List of abbreviations

ALL: adult lymphoblastic leukemia
CAGR: cancer-associated genomic regions
CLL: chronic lymphocytic leukemia
CRC: colorectal cancer
DAPI: 4',6-diamidino-2-phenylindole
EFV: efavirenz
FITC: fluorescein isothiocyanate
FRA: fragile sites
HCC: hepatocellular carcinoma
HML: human myeloid leukemia
IF: immunuofluorescence
LINE-1 (L1): long interspersed element-1
LOH: loss of heterozigosity
miRNAs: micro RNAs
RT: reverse transcriptase
UCR: ultra conserved regions
