# Supplementary material for: A tumor-promoting mechanism mediated by retrotransposon-encoded reverse transcriptase is active in human transformed cell lines
Source: Oncotarget. 2013 Oct 14;4(12):2271–87. doi: 10.18632/oncotarget.1403 (PMC3926826; doi:10.18632/oncotarget.1403)
Supplement: Supplementary file 2 [file oncotarget-04-2271-s002.pdf]

A tumor-promoting mechanism mediated by retrotransposon-encoded reverse transcriptase is active in human transformed cell lines – Sciamanna et al

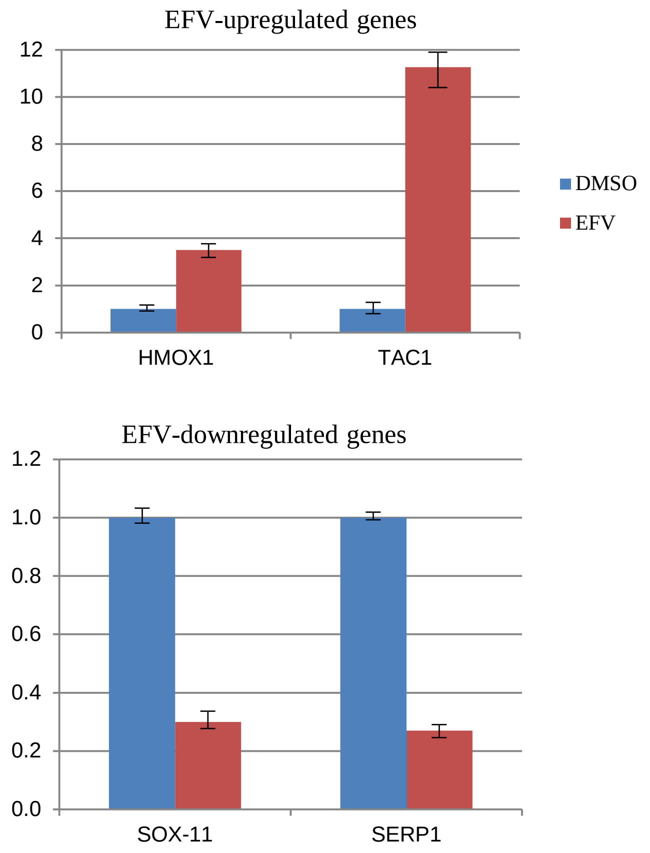

Figure S1: Real Time PCR analysis of gene expression in EFV-treated A-375 cells. The histograms represent the fold-increase or decrease in mRNA levels for the indicated genes, selected from the list in Additional file 1, in EFV-treated cells relative to levels measured in the DMSO samples (set as reference). Bars represent the standard error of the mean expression level and delimit the calculated maximum and minimum level of expression. Triplicates for each sample were analyzed in two independent experiments.

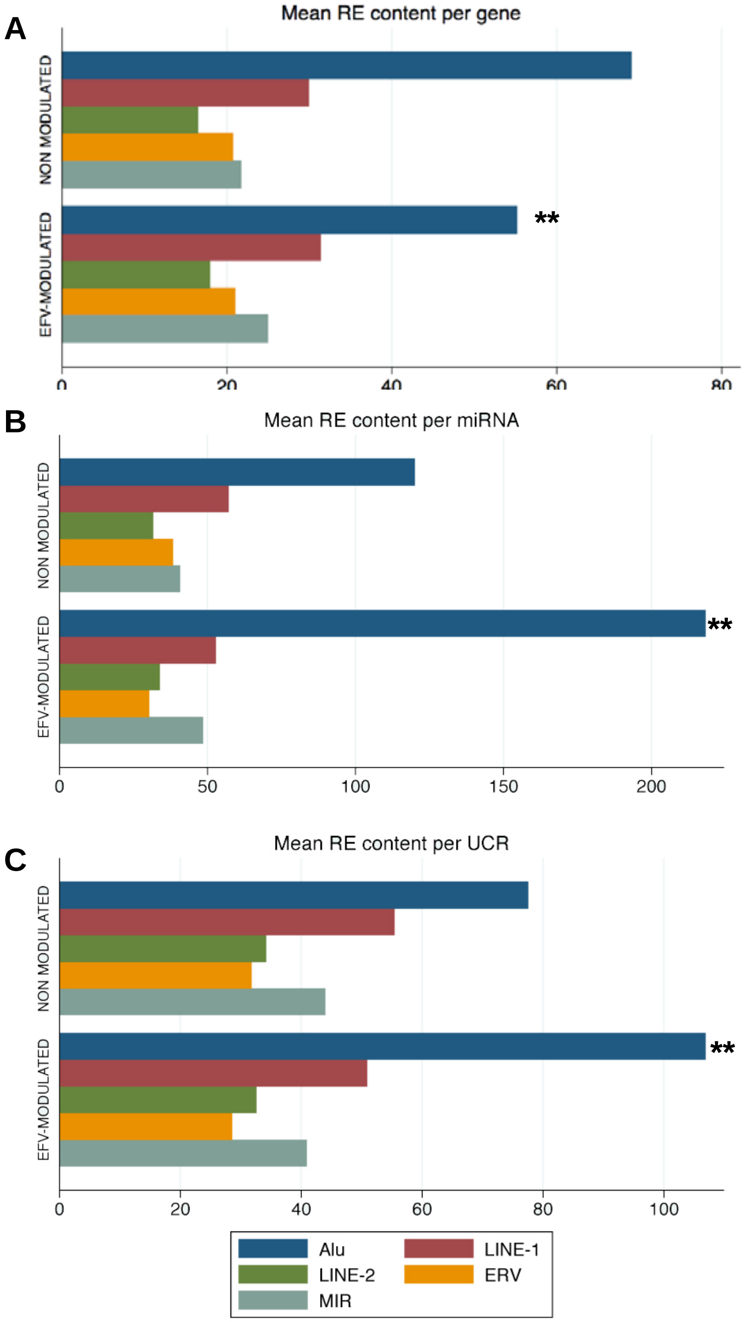
A
Mean RE content per gene
NON MODULATED
EFV-MODULATED
**
0
20
40
60
80
B
Mean RE content per miRNA
NON MODULATED
EFV-MODULATED
**
0
50
100
150
200
C
Mean RE content per UCR
NON MODULATED
EFV-MODULATED
**
0
20
40
60
80
100
Alu
LINE-1
LINE-2
ERV
MIR
